# hsa_circ_0001955 Promotes Colorectal Cancer Progression by Regulating miR-583/FGF21 Axis

**DOI:** 10.1155/2022/4288474

**Published:** 2022-05-11

**Authors:** Xuefeng Gao, Junfeng Yin, Ying Yao

**Affiliations:** ^1^Department of Gastroenterology, Affiliated Hospital of Yangzhou University, Yangzhou, Jiangsu 225000, China; ^2^Department of General Surgery, Affiliated Hospital of Yangzhou University, Yangzhou, Jiangsu 225000, China; ^3^Department of Medical Laboratory, Affiliated Hospital of Yangzhou University, Yangzhou, Jiangsu 225000, China

## Abstract

**Objective:**

Hsa_circ_0001955 presents significant upregulation in colorectal cancer (CRC) tissues. However, its role in CRC remains unclear. Thus, we attempted to clarify functions of hsa_circ_0001955 on CRC.

**Methods:**

qRT-PCR was performed to examine hsa_circ_0001955, miR-583, and FGF21 levels. Western blotting was conducted to measure FGF21 protein expression. CCK-8, flow cytometry, and Ki-67 immunohistochemical staining and TUNEL assays were conducted to assess proliferation and apoptosis *in vitro* and *in vivo*, respectively. Cell invasion and migration were assessed by Transwell assay. Tumor-bearing mouse model and HE staining were used to assess inflammatory injury. Luciferase reporter system and RNA pull-down were conducted to evaluate the regulation between miR-583 and hsa_circ_0001955 or FGF21.

**Results:**

We found that hsa_circ_0001955 showed characteristics of upregulated circRNA in CRC. Further analysis indicated that hsa_circ_0001955 elevation facilitated CRC cell malignancy *in vitro* and promoted tumor growth *in vivo*. Furthermore, hsa_circ_0001955 was a miR-583 sponge and FGF21 was directly targeted by miR-583. In addition, we found that downregulation of miR-583 promoted hsa_circ_0001955-mediated CRC cell malignancy *in vitro*. In contrast, FGF21 elevation promoted miR-583-regulated CRC cell malignancy *in vitro*.

**Conclusion:**

We demonstrated that hsa_circ_0001955 facilitated CRC progression via miR-583/FGF21 axis, suggesting that hsa_circ_0001955 may provide a novel insight for therapy of CRC.

## 1. Introduction

Colorectal cancer (CRC), one of commonly seen digestive tract malignancies, currently ranks the third among malignant tumor incidences worldwide [1]. As the way of diet changes with economic development, CRC incidence in China increased rapidly by year [2]. Its etiology is not clear due to inobvious symptoms in the early stage and high metastatic rate. These are important factors leading to its poor prognosis and high mortality [3]. Therefore, it is important for CRC research to explore its occurrence, development, and metastasis, thus to find efficacious prevention and treatment.

Circular RNAs (circRNAs) are novel subgroup of special noncoding RNAs without a free 5′-end cap and 3′-end poly (A) tail and generate the circular structure by covalent bonds. CircRNA is not affected by RNA exonuclease since its expression is stable, and it is not easily degraded [4, 5]. CircRNAs abundantly express in eukaryotic cell cytoplasm with certain organization, timing, and disease specificity [5]. Multiple studies have shown that circRNA exerts vital roles in progression of numerous tumors including CRC [6, 7]. For instance, circDDX17 was downregulated in CRC by RNA sequencing, indicating the tumor suppressor role in CRC [8]. CircGLIS2 presented upregulation in CRC and circGLIS2 overexpression promoted CRC cell growth through NF-*κ*B pathway activation [9]. In addition, several circRNAs were considered to be novel biomarkers and therapeutic targets for CRC, including circPTK2, circRNA-100876, and circ_0026344 [6, 10–12]. Taken together, these findings suggested that circRNAs are promising molecular targets for CRC treatment. However, functions of many potential circRNAs in CRC have not yet been reported.

In recent work, hsa_circ_0001955 was confirmed with high expression in CRC by whole-transcriptome analysis [13]. However, hsa_circ_0001955 function on CRC progression remains unclear. Therefore, we intended to clarify the role of hsa_circ_0001955 in CRC progression both *in vitro* and *in vivo*.

## 2. Materials and Methods

### 2.1. Patient Samples

Thirty paired CRC tissues and adjacent normal tissues were collected from Affiliated Hospital of Yangzhou University. All participants have written informed consent. This study was approved by the Ethics Committee of Affiliated Hospital of Yangzhou University.

### 2.2. Cell Culture and Transfection

CRC cancer cell lines Caco2, HT29, HCT15, HCT116, and sw620 cells and normal colorectal mucosal cell line FHC were purchased from Procell Life Science & Technology (Wuhan, Hubei, China) and cultured in DMEM medium (Gibco) supplemented with 10% fetal bovine serum (Gibco) at 37°C in a humidified atmosphere containing 5% CO_2_. Different cells were seeded in 12-well plates and transfected with Over-hsa_circ_0001955, sh-hsa_circ_0001955, Over-FGF21, sh-FGF21, miR-583 mimics, and miR-583 inhibitors using Lipofectamine 2000 (Invitrogen) in accordance with the manufacturer's instructions when the cell confluence reached 70-90%. And then the cell was analyzed 48 h after transfection.

### 2.3. RNA Isolation and qRT-PCR Analysis

TRIzol (Invitrogen) was used to isolate total RNA from CRC cells or tissue according to the manufacturer's instructions. miRNA was extracted and purified using an mirVana™ miRNA isolation kit (Invitrogen). Then, the RNA was reverse-transcribed into cDNA. qPCR was conducted by miRNA or SBGY qPCR master mix under an ABI7500 machine. U6 and GAPDH were used as an internal control for miRNA and circRNA or mRNA, respectively. The amplification processes are as follows: 95°C for 5 min, 95°C for 10 s, 60°C for 30 s, and 72°C for 30 s with 40 cycles. The expression levels of the target genes were calculated using 2^−*ΔΔ*CT^ method. All the primer sequences were synthesized from Promega (Madison, WI, USA) and the sequences of the primers were as follows: circ_0001955: forward 5′-GATCATTCATCGGCTACCAA-3, reverse 5′-ATCATTTACTGGACTAGGTACAGC-3′; miR-583: forward 5′-GGATTGGCCACAATGGGT-3′, reverse 5′-GTCATATCGATTGCCTGT-3′; FGF21: forward 5′-TTACGATTACCTACGAT-3′, reverse 5′-CCGATCCATGACTGAAC-3′; U6: forward 5′-CTCGCTTCGGCAGCACA-3′, reverse 5′-AACGCTTCACGAATTTGCGT-3′; GAPDH: forward 5′-CTGGGCTCACTGAGCACC-3′, reverse 5′-AAGTGGTCGTTGAGGGCAATG-3′.

### 2.4. RNase R and Actinomycin D Treatment

RNase R enzyme (Termo Fisher, Grand Island, NY, USA) digestion and actinomycin D (Sigma, Missouri, USA) treatment were used to detect the stability of hsa_circ_0001955. For RNase R digestion, the total hsa_circ_0001955 extracted from HCT116 was added or not added to the RNase R enzyme (20 U/*μ*L), incubated at 37°C for 30 min and then at 70°C for 10 min to inactivate the RNase R. This was followed by RT-qPCR detection. For the actinomycin D treatment, HCT116 were added with actinomycin D at a final concentration of 1 *μ*g/mL at 0, 4, 8, 12, and 24 h, and RNA was collected for RT-qPCR analysis.

### 2.5. Western Blot

Proteins were extracted by RIPA buffer and equal proteins were load onto a sodium dodecyl sulfate–polyacrylamide gel and then transferred to a PVDF membrane (Millipore, USA). The membrane was incubated with primary antibody anti-FGF21 (ab171941, 1 : 1000, Abcam, Cambridge, UK) and anti-GADPH (ab181602, 1 : 10000, Abcam, Cambridge, UK) after being washed with TBST buffer and blocked with BSA for 24 h. The proteins were then incubated with a secondary antibody (ab96899, 1 : 1000, Abcam, Cambridge, UK). Finally, the proteins were visualized using ECL-plus reagents and analyzed with Image J.

### 2.6. Subcellular Localization Assay

Cytoplasm and nucleus of HCT116 were isolated using a PARIS™ kit (Thermo Fisher Scientific, Madison, WI, USA) following the manufacturer's protocols. Hsa_circ_0001955 from the isolated cytoplasmic and nuclear fractions was reverse-transcribed and used for PCR after purification and DNase I treatment. GAPDH served as the endogenous control for the cytoplasm, and MALAT1 for the nucleus.

### 2.7. FISH Assay

The location of hsa_circ_0001955 in HCT116 was detected using FISH kit (RiboBio) following the manufacturer's protocol. The cells (1 × 10^3^ cells for each group) were subjected to prehybridization solution for 30 min. Then, probes were dissolved at a concentration of 20 *μ*mol/L and subjected to slides and hybridized for 12 hours. Next, saline sodium citrate was used to wash slides. DAPI was added for 15 min. A confocal microscopy was taken to analyze results.

### 2.8. RNA Pull-Down Assay

The biotinylated of miR-583 was synthesized by Promega (Madison, WI, USA) and the related oligonucleotides were transfected into melanoma cells. The lysates of cells were cultivated with M-280 streptavidin magnetic beads (Invitrogen). circRNA level in the bound RNA was detected by qRT-PCR. The biotinylated mutant miR-583 and miR-583 NC were used as control.

### 2.9. CCK-8 Cell Viability Assay and Flow Cytometry

Cells were cultured in 96-well plates and cultured for 24, 48, and 72 h. Then, the viability was detected using CCK-8 Kit [14]. The absorbance value was examined at 450 nm on a microplate reader. For apoptosis assay, HCT116 cells with different treatment were cultured for 48 h. Then, the cells were collected and labeled with Annexin V-FITC and PI (propidium iodide; BD Biosciences) for 30 min. Cells with FITC^+^ and PI^−^ were considered early apoptotic. Cells with FITC^+^ and PI^+^ were considered late apoptotic. The calculation was expressed as the proportion of early and late apoptotic cells. Finally, the apoptotic cells were quantified by a fluorescence-activated cell sorting flow cytometer (BD Biosciences).

### 2.10. Transwell Assay

A polycarbonate membrane Boyden chamber was used to detect the migration and invasion of HCT116 cells with different treatment. Briefly, the cells were treated with trypsin and resuspended in DMEM and then added to an 8 *μ*m-pore polycarbonate membrane Boyden chamber to detect migration. In another group with the same treatment, 25 mg of Matrigel (BD Biosciences, CA, USA) was added to detect invasion. DMEM with 10% FBS was added to all the lower chambers. After 24 hours of incubation, cells were treated with crystal violet staining (1 × 10^3^ cells for each group). Penetrating cells were calculated repeatedly 3 times with 5 randomly picked fields.

### 2.11. Luciferase Assay

Hsa_circ_0001955 or FGF21 sequence with wild-type or mutant binding site was synthesized from Promega (Madison, WI, USA) and cloned into the dual-luciferase reporter pmirGLO vector. And then the vectors were cotransfected with the miR-583 mimic or miR-583 NC into HCT116. Luciferase activity was estimated using a Dual-Luciferase Reporter assay system (Promega) after 48 h of transfection. Renilla luciferase activity was normalized against firefly luciferase activity.

### 2.12. Xenograft Tumor Model

For the xenograft tumor model, 4-week-old BALB/c nude mice were purchased from Charles River (Beijing, China) and randomly divided into 3 groups (*n* =5, each), including NC, Over-circRNA, and sh-CircRNA groups. 5 × 10^6^ HCT116 cells were inoculated subcutaneously into nude mice and the tumor volumes were measured once every 7 days by digital calipers using *V* = *D* × *d*^2^/2 (*D* = long axis of the tumor and *d* = short axis of the tumor) [15] for a total of 28 days. After 4 weeks, the mice were sacrificed, and the weights of the tumors were measured. The tumor tissues were harvested, and the pathological changes were detected by hematoxylin and eosin (H&E) staining. Tumor tissues from mice were formalin-fixed and paraffin-embedded, cut into 4-*μ*m-thick sections, and used for H&E staining. The changes in histology were assessed under a light microscope. The animal assay was approved by the Committee for Animal Care and Use of Affiliated Hospital of Yangzhou University.

### 2.13. Ki-67 Immunostaining

Normal and neoplastic tissue proliferation was detected using a rabbit monoclonal primary antibody CONFIRM® anti-Ki-67 (30-9) (790-4286, Ventana Medical Systems, Tucson, Arizona, USA), which serves directly against the C-terminal portion of the Ki-67 antigen. The applied antibody for routine diagnostic IHC obtained FDA approval (510 k) for IVD (in vitro diagnostic) use. Brown nuclear (DAB) staining in tumor cells was identified as Ki-67 positive cells. The ARIOL imaging system (Applied Imaging Corp., San Jose, CA, USA) and light microscope quantified Ki-67 index.

### 2.14. Terminal Deoxynucleotidyl Transferase-Mediated dUTP Nick-End-Labeling (TUNEL) Assay

TUNEL detection kit (Promega) was used for the detection of cell apoptosis in tumor tissues. Briefly, the tumor sections were deparaffinized, dehydrated, and treated with 20 *μ*g/mL Proteinase K for 20 min. Then, the sections were incubated with TUNEL reaction mixture for 1 h at 37°C after rinsing with 0.3% Triton X-100 for 10 min. After that, the sections were treated with HRP conjugated streptavidin at 37°C for 30 min and mixed with 0.04% DAB and 0.03% H_2_O_2_ at room temperature for 8-12 min. The slices were counter-stained with hematoxylin. The changes in histology were assessed under a light microscope. Cells with blue granules in the nucleus were regarded as positive for TUNEL.

### 2.15. Statistical Analysis

Statistical analysis was conducted using SPSS 21.0 (IBM, SPSS, Chicago, IL, USA). The differences between two or more groups were detected using Student's *t* test and one-way ANOVA and *p* <0.05 was considered to be significant.

## 3. Results

### 3.1. hsa_circ_0001955 Shows Upregulation in CRC Tissues and CRC Cells

**Figure 1:**
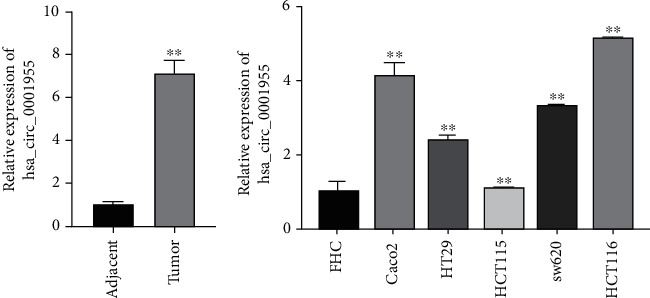
hsa_circ_0001955 was upregulated in the CRC tissues and CRC cells. (a) qRT-PCR detected hsa_circ_0001955 level in CRC tissues, ∗∗*p* < 0.01, versus adjacent group; (b) qRT-PCR detected hsa_circ_0001955 level in human normal colorectal mucosal cell line FHC and CRC cell lines Caco2, HT29, HCT15, HCT116, and sw620 cells, ∗∗*p* < 0.01, versus FHC group.

hsa_circ_0001955 was reported to have high expression in CRC by whole-transcriptome analysis. In order to further confirm the expression level of hsa_circ_0001955 in CRC tissues, qRT-PCR was performed. hsa_circ_0001955 showed way higher expression in the CRC tissues than the controls (Figure 1(a)). Furthermore, the expression of hsa_circ_0001955 was significant in Caco2, HT29, HCT15, HCT116, and sw620 cells compared to FHC cells (Figure 1(b)), among which HCT116 cells showed the highest expression. Therefore, HCT116 cells were used for further study. These results indicated that hsa_circ_0001955 presented elevation in CRC tissues and cells to promote CRC progression.

### 3.2. Characterization of hsa_circ_0001955 in CRC

**Figure 2:**
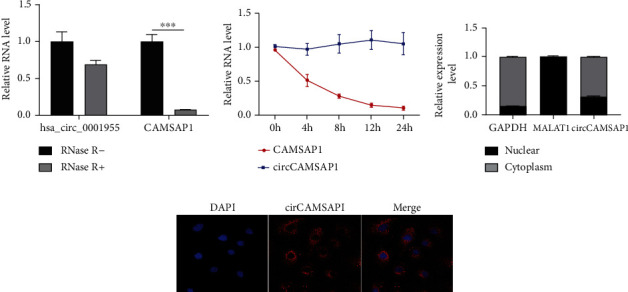
Characterization of hsa_circ_0001955 in CRC. (a and b) qRT-PCR detect the expression of hsa_circ_0001955 level under RNase R treatment, ∗∗∗*p* < 0.001, versus RNase R-group; (c) qRT-PCR detected has_circ_0001955 subcellular localization; (d) fluorescence in situ hybridization (FISH) assay detected has_circ_0001955 subcellular localization.

hsa_circ_0001955 level was not affected under RNase R and actinomycin D compared with linear CSNK1G1 mRNA (Figure 2(a)), suggesting that hsa_circ_0001955 (circCAMSAP1) had more stability than linear mRNA (Figure 2(b)). Moreover, subcellular localization (http://lncatlas.crg.eu) and FISH illustrated that hsa_circ_0001955 mostly located in the cytoplasm (Figures 2(c) and 2(d)).

### 3.3. Overexpression of hsa_circ_0001955 Promotes CRC Cell Malignancy

**Figure 3:**
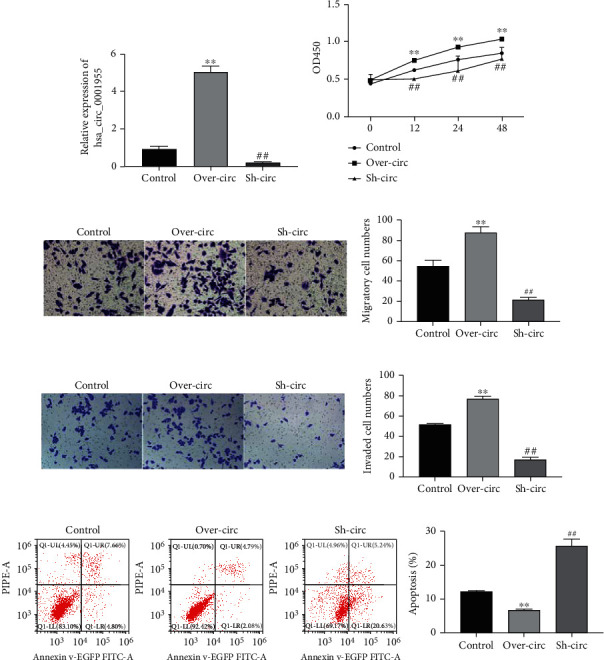
Overexpression of hsa_circ_0001955 promoted CRC cell proliferation, migration, and invasion and inhibited apoptosis in vitro. (a) qRT-PCR detected hsa_circ_0001955 level under transfection with Over-hsa_circ_0001955 or sh-hsa_circ_0001955 vector, respectively; (b) CCK-8 assay detected HCT116 cell proliferation affected by hsa_circ_0001955; (c and d) Transwell assay detected HCT116 cell migration and invasion affected by hsa_circ_0001955, respectively. (e) flow cytometry detected HCT116 cell apoptosis affected by hsa_circ_0001955. ∗∗*p* < 0.01, ##*p* < 0.01, versus control group.

To clarify the role of hsa_circ_0001955 in CRC, we conducted loss and gain function of hsa_circ_0001955. hsa_circ_0001955 was successfully overexpressed or suppressed in HCT116 cells (Figure 3(a)). CCK-8 depicted that the overexpression of hsa_circ_0001955 notably promoted cell proliferation while knockdown of it showed the opposite results (Figure 3(b)). Transwell assay indicated that the migration and invasion of HCT116 cells markedly increased with the overexpression of hsa_circ_0001955, while the HCT116 cells treated with sh-hsa_circ_0001955 showed the opposite (Figures 3(c) and 3(d)). Additionally, flow cytometry demonstrated the decreased apoptosis rate with Over-hsa_circ_0001955 and the opposite trend with sh-hsa_circ_0001955 (Figure 3(e)). These results revealed that the upregulation of hsa_circ_0001955 promoted CRC cell growth in vitro.

### 3.4. hsa_circ_0001955 Is a Molecular Sponge of miR-583

**Figure 4:**
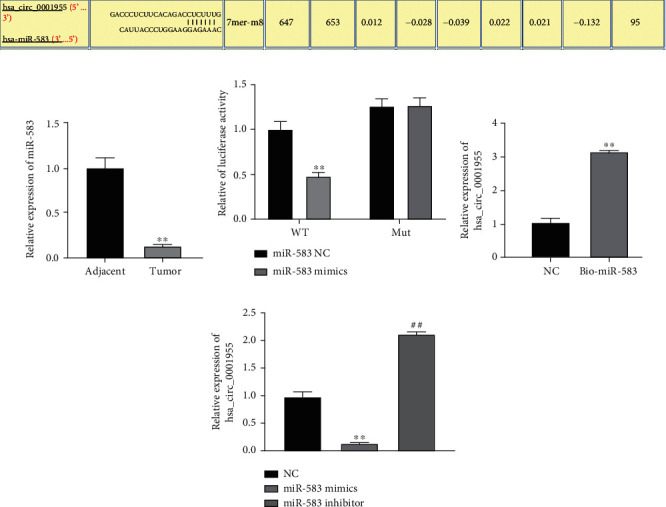
hsa_circ_0001955 is a molecular sponge of miR-583. (a) CircInteractome predicted downstream of hsa_circ_0001955; (b) qRT-PCR analysis detected miR-583 level in the CRC tissues, ∗∗*p* < 0.01, versus adjacent group; (c) dual-luciferase reporter assays detected relationship of miR-583 and hsa_circ_0001955. ∗∗*p* < 0.01, versus NC group; (d) RNA pull-down detected relationship between miR-583 and hsa_circ_0001955, ∗∗*p* < 0.01, versus NC group; (e) RT-qPCR detected t hsa_circ_0001955 level affected by miR-583, ∗∗*p* < 0.01, ##*p* < 0.01, versus NC group.

CircRNA could act as molecular sponge of miRNA to regulate gene expression. CircInteractome estimated downstream miRNA of hsa_circ_0001955. miR-583 showed highest binding score between miR-583 and hsa_circ_0001955 (Figure 4(a)). qRT-PCR illustrated that miR-583 level presented decrease in the CRC tissues (Figure 4(b)). Luciferase reporter assays revealed that upregulation of miR-583 inhibited the luciferase activity of hsa_circ_0001955 containing the wild-type binding site. However, luciferase activity was not affected by miR-583 mimics cotransfected with hsa_circ_0001955 containing mutant binding site in HCT116 cells (Figure 4(c)). By using a bio-miR-583 probe, RNA pull-down assay was conducted to analyze the association between miR-583 and hsa_circ_0001955. It was found that hsa_circ_0001955 has high expression in the enrichment of miR-583, suggesting that hsa_circ_0001955 may bind to miR-583 (Figure 4(d)). In addition, qRT-PCR demonstrated that hsa_circ_0001955 level presented decrease in the miR-583 mimic group while increase under miR-583 depletion in HCT116 cells (Figure 4(e)). These results suggested that hsa_circ_0001955 sponged miR-583.

### 3.5. Downregulation of miR-583 Promotes CRC Cell Malignancy In Vitro Sponging by hsa_circ_0001955

**Figure 5:**
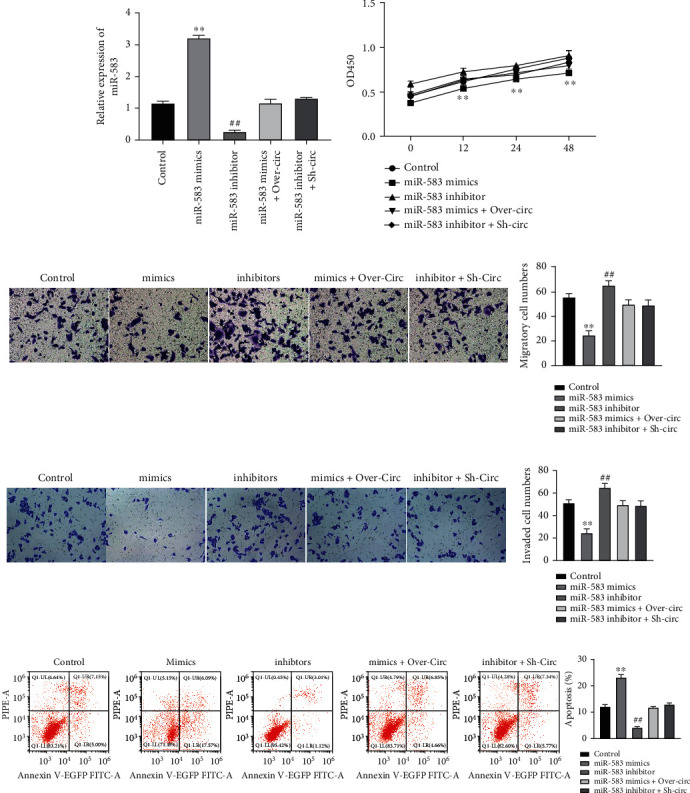
Downregulation of miR-583 promoted CRC cell proliferation, migration, and invasion and inhibited apoptosis in vitro sponging by hsa_circ_0001955. (a) qRT-PCR detected miR-583 level in HCT116 cells; (b) CCK-8 detected cell proliferation affected by miR-583 and reversed by hsa_circ_0001955; (c and d) Transwell assay detected HCT116 cell migration and invasion affected by miR-583 and reversed by hsa_circ_0001955; (e) flow cytometry detected apoptosis affected by miR-583 and reversed by hsa_circ_0001955; ∗∗*p* < 0.01, ##*p* < 0.01, versus NC group.

To investigate miR-583 role underlying CRC, miR-583 mimics or inhibitors was used to treat HCT116 cells. miR-583 level was successfully overexpressed or suppressed in HCT116 cells (Figure 5(a)). CCK-8 showed the inhibited cell proliferation under miR-583 upregulation and opposite trend under miR-583 downregulation in the HCT116 cells (Figure 5(b)). Transwell assay indicated that the numbers of migratory and invaded HCT116 cell markedly decreased under miR-583 elevation and increased under miR-583 depletion (Figures 5(c) and 5(d)). Flow cytometry demonstrated that apoptosis increased under miR-583 overexpression while decreased under miR-583 downregulation (Figure 5(e)). Additionally, hsa_circ_0001955 reversed the impact of miR-583 on CRC cells, including proliferation, migration, invasion, and apoptosis (Figures 5(b)–5(e)). Collectively, the sponging of miR-583 with hsa_circ_0001955 downregulated its expression and promoted CRC cell malignancy *in vitro*.

### 3.6. FGF21 Is Directly Targeted by miR-583

**Figure 6:**
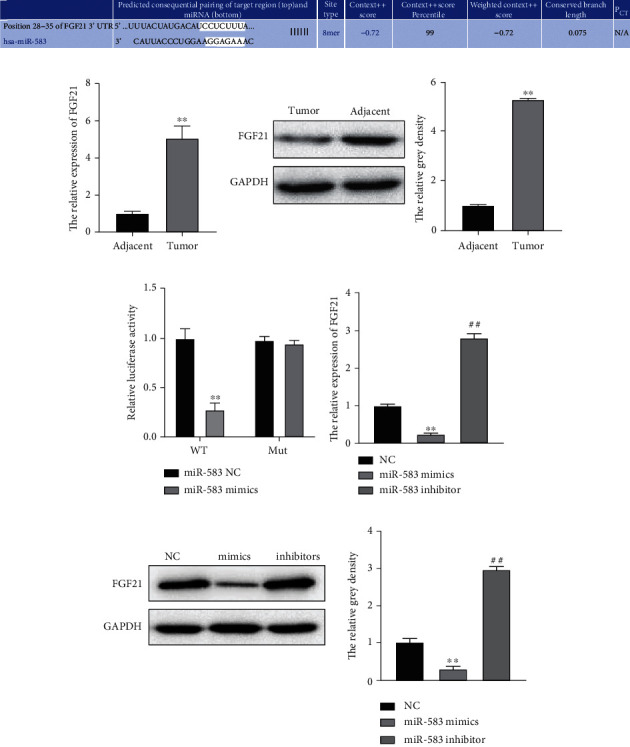
FGF21 is directly targeted by miR-583. (a) TargetScan predicted targets of miR-583; (b and c) qRT-PCR and western blotting detected FGF21 level in CRC tissues, ∗∗*p* < 0.01, versus adjacent group; (d) dual-luciferase reporter assays detected regulationship of miR-583 and hsa_circ_0001955. ∗∗*p* < 0.01, versus NC group; (e and f) RT-qPCR and western blotting detected FGF21 level affected by miR-583, ∗∗*p* < 0.01, ##*p* < 0.01, versus NC group.

TargetScan predicted targeted molecule of miR-583. FGF21 is a putative target of miR-583 possessing binding sequence at position 28-35 of 3′UTR (Figure 6(a)). qRT-PCR and western blotting indicated that FGF21 showed dramatical upregulation in the CRC tissues relative to controls (Figures 6(b) and 6(c)). Luciferase reporter assay demonstrated that miR-583 upregulation repressed luciferase activity of FGF21 3′UTR-WT in HCT116 cells. However, luciferase activity of FGF21 mutation was not affected in HCT116 cells (Figure 6(d)). Additionally, RT-qPCR and western blotting demonstrated FGF21 downregulation with miR-583 overexpressed and FGF21 upregulation with miR-583 depleted in HCT116 cells (Figures 6(e) and 6(f)). It was suggested that FGF21 was negatively modulated by miR-583.

### 3.7. FGF21 Overexpression Promotes CRC Cell Malignancy In Vitro Regulated by miR-583

**Figure 7:**
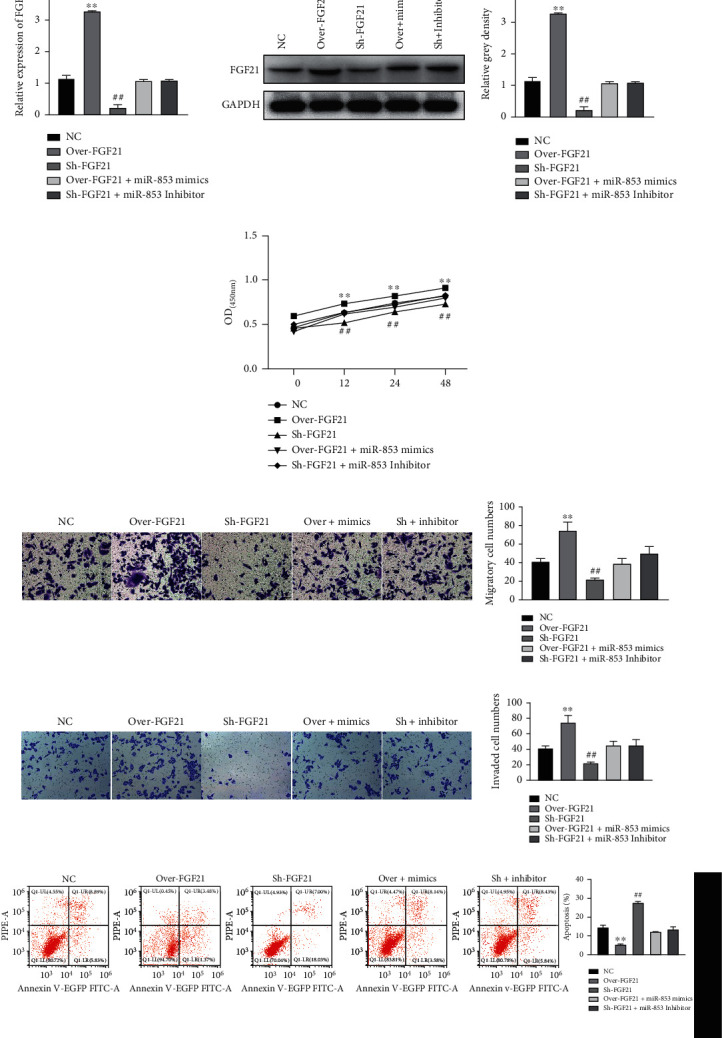
Overexpression of FGF21 promoted CRC cell proliferation, migration, and invasion and inhibited apoptosis in vitro regulated by miR-583. (a and b) qRT-PCR and western blotting detected FGF21 level in HCT116 cells; (c) CCK-8 detected HCT116 cell proliferation affected by FGF21 and reversed by miR-583; (d and e) Transwell assay detected HCT116 cell migration and invasion affected by FGF21 and reversed by miR-583; (f) flow cytometry detected HCT116 cell apoptosis affected by FGF21 and reversed by miR-583. ∗∗*p* < 0.01, ##*p* < 0.01, versus NC group.

To clarify the role of FGF21 in CRC, loss and gain function of FGF21 was performed. qRT-PCR and western blotting illustrated successful overexpression or inhibition of FGF21 in HCT116 cells (Figures 7(a) and 7(b)). CCK-8 depicted the proliferation increased with FGF21 overexpressed and decreased with FGF21 silenced in HCT116 cells (Figure 7(c)). Transwell assay indicated that the migration and invasion of HCT116 cell markedly increased with FGF21 overexpressed and decreased with FGF21 silenced (Figures 7(d) and 7(e)). Additionally, flow cytometry demonstrated that apoptosis dramatically decreased with the overexpression of FGF21 while increased with the suppression of FGF21 (Figure 7(f)). On contrast, the influence of FGF21 on HCT116 cell proliferation/migration/invasion/apoptosis was reversed by miR-583 (Figures 7(c)–7(f)). These results revealed that the elevation of FGF21 promoted malignancy of CRC cells *in vitro*, which was regulated by miR-583.

### 3.8. hsa_circ_0001955 Overexpression Promotes CRC Tumor Growth

**Figure 8:**
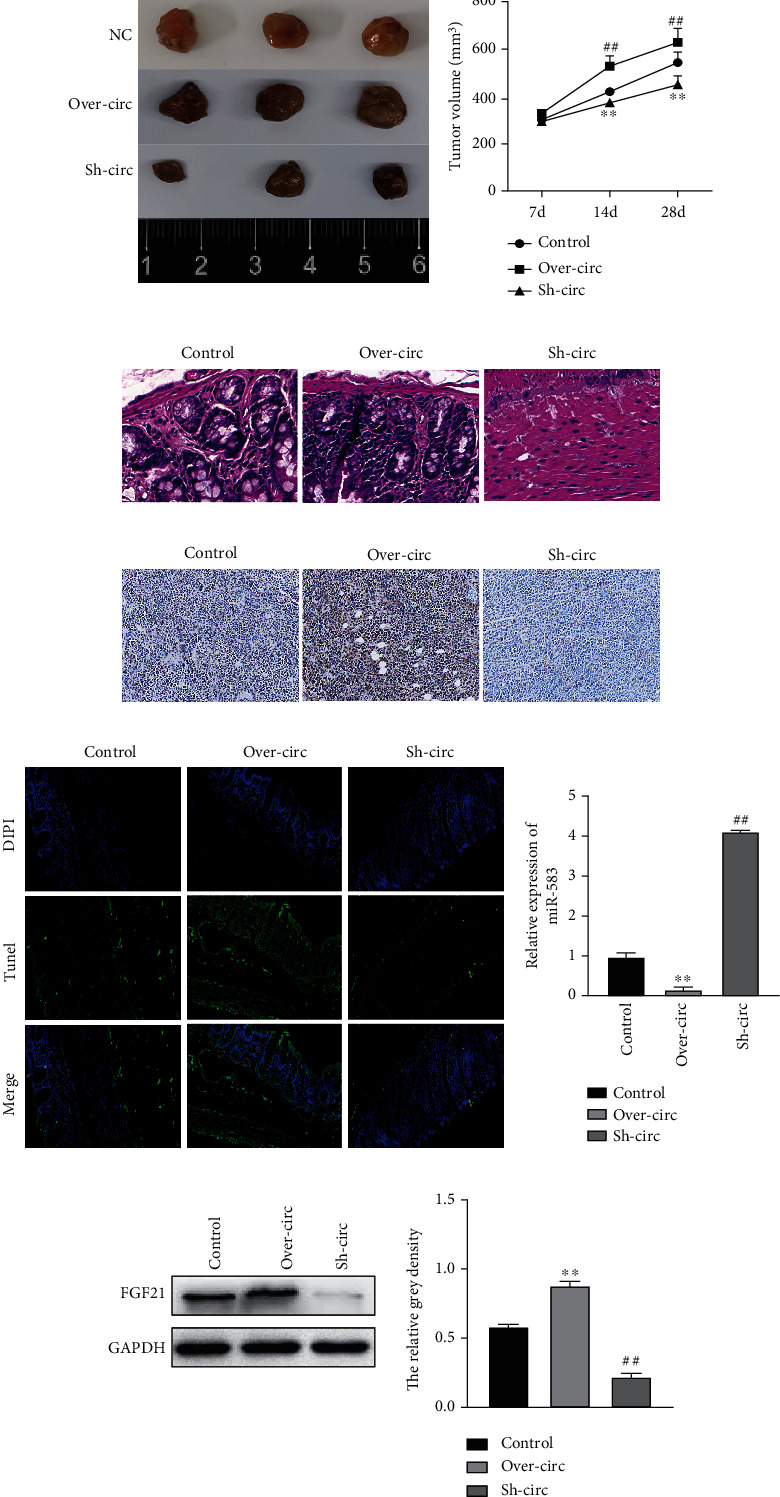
Overexpression of hsa_circ_0001955 promoted CRC tumor growth *in vivo.* (a) Tumor size affected by hsa_circ_0001955; (b) H&E staining detected inflammation affected by hsa_circ_0001955; (c) Ki-67 immunohistochemistry detected cell proliferation affected by hsa_circ_0001955; (d) TUNEL assay detected cell apoptosis affected by hsa_circ_0001955; (e) qRT-PCR detect miR-583 level affected by hsa_circ_0001955; (f) western blotting detected FGF21 level affected by hsa_circ_0001955, respectively. ∗∗*p* < 0.01, ##*p* < 0.01, versus control group.

The role of hsa_circ_0001955 was also verified in a tumor-bearing nude mice with injection of HCT116 subcutaneously. Tumor size increased with hsa_circ_0001955 elevation and decreased with hsa_circ_0001955 depletion (Figure 8(a)). H&E staining showed that the inflammatory injury was obvious with hsa_circ_0001955 elevation while the inflammatory injury was alleviated with hsa_circ_0001955 depletion (Figure 8(b)). Ki-67 immunohistochemistry indicated that the overexpression of hsa_circ_0001955 promoted cell proliferation while the knockdown of hsa_circ_0001955 had an opposite impact (Figure 8(c)). TUNEL assay illustrated that the overexpression of hsa_circ_0001955 repressed cell apoptosis while the knockdown of hsa_circ_0001955 promoted cell apoptosis (Figure 8(d)). Additionally, miR-583 showed downregulation in Over-hsa_circ_0001955 group and upregulation in sh-hsa_circ_0001955 group (Figure 8(e)). In contrast, FGF21 level showed the opposite results compared to miR-583 expression changes (Figure 8(f)). To sum up, the elevation of hsa_circ_0001955 promoted CRC tumorigenesis *in vivo*.

## 4. Discussion

CRC, a common digestive system malignancy, poses a severe threat to human health. CRC progression is a multivariate and multistep process, of which the pathogenesis has not yet been fully understood [16]. Nowadays, CRC 5-year survival is still below 60%. The clinical efficacy evaluation and prognosis monitoring need to be further improved [17]. Thus, it is of huge importance to explore hidden factors affecting CRC progression. Herein, hsa_circ_0001955 exhibited high expression in CRC, which is consistent with previous research. Mechanically, we found that hsa_circ_0001955 facilitated CRC malignancy via miR-583/FGF21 axis, suggesting that hsa_circ_0001955 was a promising target for CRC treatment.

CircRNA, characterized by great stability and conservation among species, as well as tissue specificity, has been regarded by researchers as promising biological targets for clinical diagnosis and therapy [6, 18]. CircRNAs play vital roles in CRC progression through modulating various biological behaviors, including proliferation, migration, invasion, and drug resistance. Previously, it has been found that hsa_circ_0001955 exhibits high expression in CRC samples by whole-transcriptome analysis [13]. Consistent with that result, we found that hsa_circ_0001955 expressed highly in CRC tissues and cells, suggesting that it may serve as an oncogene of CRC. Previous reports have proved that hsa_circ_0001955 promotes hepatocellular carcinoma [19]. Ding et al. indicated that hsa_circ_0001955 facilitates HCC cell growth *in vitro* via regulating miR-145-5p/NRAS axis [20]. Nevertheless, the effect of hsa_circ_0001955 on CRC remains unclear. In our study, the overexpression of hsa_circ_0001955 promoted CRC cell growth and suppressed apoptosis *in vitro* and tumor growth *in vivo*. Collectively, hsa_circ_0001955 promotes CRC progression.

CircRNAs adsorb miRNAs via functioning as miRNA molecular sponges [21, 22], thus regulating gene expression, and are widely involved in life activities and tumor progression [23–25]. Increasing numbers of studies also revealed the sponging of circRNAs with miRNAs regulates CRC progression. For instance, hsa_circRNA_103809, with low expression in CRC, suppresses CRC cell proliferation and migration via regulating miR-532-3p/FOXO4 axis [26]. CircRNA_0000392 promotes CRC progression via PIK3R3/AKT axis by sponging miR-193a-5p [27]. Herein, we found that hsa_circ_0001955 serves as a sponge of miR-583. There is already research demonstrating miR-583 presents decreased level in HCC tissues through circRNA-miRNA-mRNA pattern [28]. Moreover, miR-583 has been found to repress the proliferation and invasion of prostate cancer cells via modulating JAK1 [29]. Herein, the expression of miR-583 sharply decreased in CRC tissues and cells. Further analysis showed that the overexpression of miR-583 promoted CRC cell proliferation/migration/invasion and suppressed apoptosis. Meanwhile, hsa_circ_0001955 upregulation counteracted the effect of miR-583 on malignancy. Taken together, hsa_circ_0001955 promotes CRC progression via sponging miR-583.

Additionally, we found that fibroblast growth factor 21 (FGF21) is the target of miR-583. It has been found that FGF21regulates various diseases, such as pulmonary diseases, obesity, and cancers [30, 31]. Recently, Yu et al. found that FGF21 promotes NSCLC progression via SIRT1/PI3K/AKT pathway [32]. In CRC, Qian et al. indicated that FGF21 might act as a circulating biomarker in multistaged colorectal carcinogenesis [33]. Sophia et al. also demonstrated that FGF21 was one of CRC biomarkers of risk prediction and diagnosis [34]. Herein, the elevation of FGF21 facilitated CRC cell proliferation/migration/invasion and repressed apoptosis. However, miR-583 upregulation reversed the changes of CRC cellular behavior brought by FGF21 elevation. Taken together, hsa_circ_0001955 promotes CRC progression via miR-583/FGF21 axis.

There remain a number of limitations to the present study. Only *in vitro* studies were performed to detect the binding mechanism. Ideally, more further studies in animal models of CRC should be performed. Furthermore, whether there exist other miRNAs or mRNAs interfering or promoting the whole modulating process remains unclear. Future investigations should focus on deepening this finding into the detailed interaction between miR-583 and hsa_circ_0001955 in CRC both *in vitro* and *in vivo*, to search for novel diagnostic and prognostic markers.

## 5. Conclusion

In conclusion, hsa_circ_0001955 shows circRNA characteristics which exhibited upregulation in CRC tissues and cells. Mechanically, hsa_circ_0001955 promotes CRC progression via miR-583/FGF21 axis, suggesting that hsa_circ_0001955 may serve as a therapeutic target of CRC.

## Data Availability

All of the data archiving will be made available on reasonable request and the correspondence responsible to the data.
